# P-1391. Mortality Risk by Site of Extrapulmonary Tuberculosis in India: A National Cohort Analysis

**DOI:** 10.1093/ofid/ofaf695.1578

**Published:** 2026-01-11

**Authors:** Urvashi B Singh, Kevin DiBona, Sanjay K Mattoo, Raghuram Rao, Nishant Kumar, Aparna Chaudhry, Pranay Sinha

**Affiliations:** All India Institute of Medical Sciences ,New Delhi, New Delhi, Delhi, India; Boston Medical Center, Boston, Massachusetts; Ministry of Health and Family Welfare, Government of India, New Delhi, Delhi, India; Indian Ministry of Health & Family Welfare, New Delhi, Delhi, India; Ministry of Health and Family Welfare, Government of India, New Delhi, Delhi, India; Wadhwani Institute for Artificial Intelligence, New Delhi, Delhi, India; Boston University, Boston, Massachusetts

## Abstract

**Background:**

India accounts for 26% of the global tuberculosis (TB) burden and 29% of TB-related deaths. Extrapulmonary TB (EPTB) makes up 15–24% of TB in India, yet mortality risk by anatomical site is poorly characterized.

**Figure 1:**
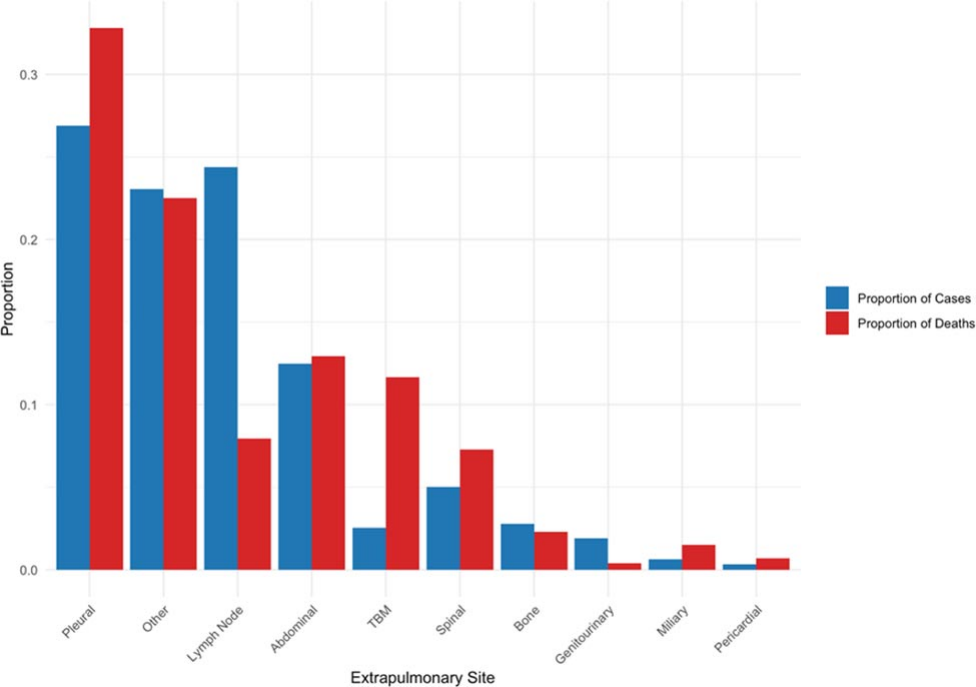
Relative prevalence and contribution to Mortality

**Figure 2:**
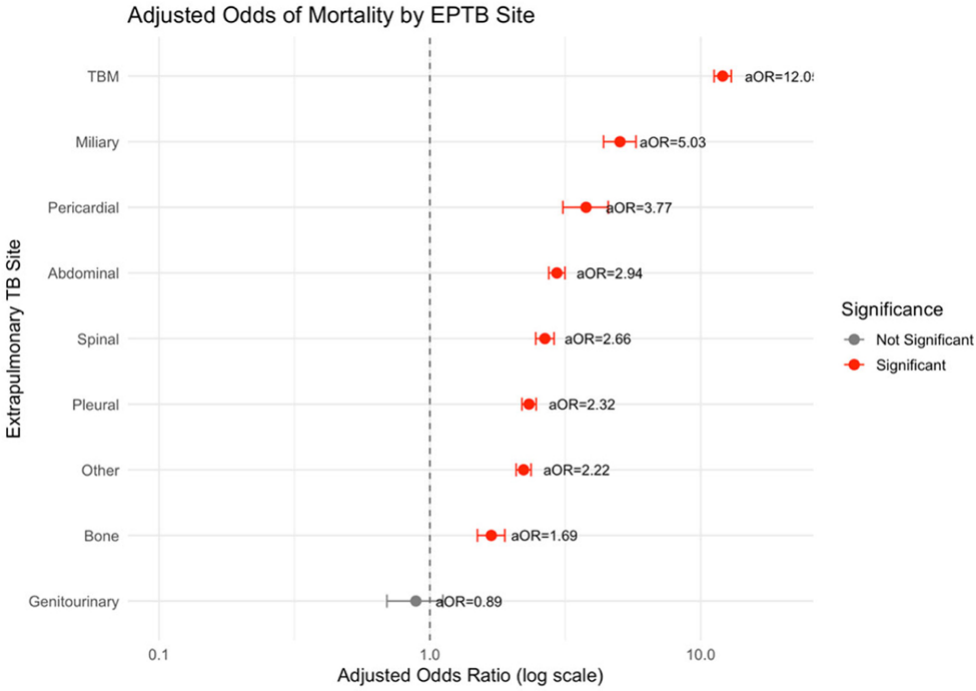
Adjusted odds ratio for mortality for different EP sites compared to lymph node TB calculated by controlling for age, sex, body mass index, HIV status, diabetes, tobacco, and alcohol use.

**Methods:**

We analyzed data from adults (≥15 years) with EPTB reported to India’s national TB database between September 2022 and December 2024. We assessed site-specific mortality using multivariable logistic regression to estimate adjusted odds ratios (aORs) for death, controlling for age, sex, body mass index (BMI), HIV status, diabetes, tobacco use, and alcohol use, with lymph node TB as the reference category. We also conducted site-stratified analyses to evaluate the association of HIV, diabetes, alcohol use, and tobacco use with mortality within each EPTB site, adjusting for age, sex, and BMI.

**Figure 3:**
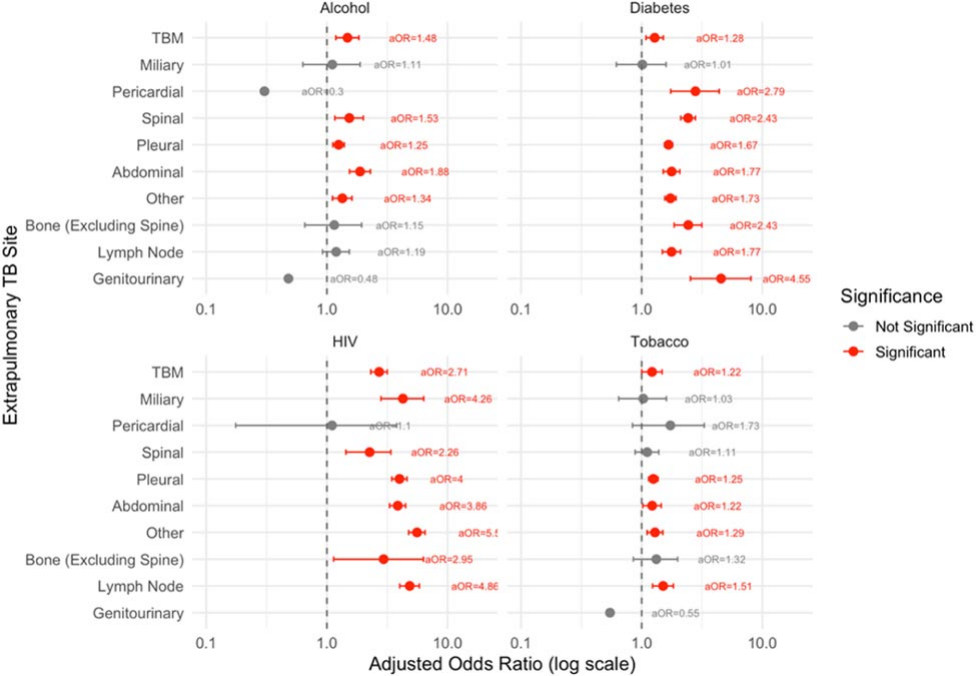
Adjusted Odds of Mortality by Site of Extrapulmonary Tuberculosis (EPTB) and Comorbidity.

**Results:**

Among 1,029,599 persons with EPTB, 777,512 (75.5%) had outcome data; 23,892 (3.1%) died. Those who died were older (mean 53 vs. 37 years), more often male (62.5% vs. 48.5%), and more likely HIV-positive (6.8% vs. 1.6%). The most common sites were pleural (20.4%), lymph node (18.5%), and abdominal (9.5%). Pleural TB accounted for one-third of EPTB deaths. (Figure 1) Tuberculous meningitis (TBM) accounted for just 3.2% of cases but 14.5% of EPTB deaths. TBM carried the highest mortality risk (aOR 12.05, 95% CI: 11.21–12.97), followed by miliary (5.03, 4.38–5.77), and pericardial (3.77, 3.10–4.56). (Figure 2) HIV infection was consistently associated with increased mortality across nearly all extrapulmonary TB (EPTB) sites, with the highest adjusted odds observed in “Other” (aOR 5.58), lymph node (aOR 4.86), and miliary TB (aOR 4.26). (Figure 3) Diabetes was also strongly associated with higher mortality in genitourinary (aOR 4.55), pericardial (aOR 2.79), and spinal TB (aOR 2.43)—while the effects of alcohol and tobacco use were more smaller and less-consistent across sites.

**Conclusion:**

This is the largest EPTB cohort to date, and it shows that mortality risk varies widely across EPTB sites. Pleural TB, TBM, miliary TB, spinal TB, and pericardial TB account for a disproportionate share of EPTB deaths and require prioritized diagnosis and treatment. EPTB mortality risk is markedly elevated in persons with HIV or diabetes.

**Disclosures:**

All Authors: No reported disclosures

